# Cleaner personality and client identity have joint consequences on cleaning interaction dynamics

**DOI:** 10.1093/beheco/arz007

**Published:** 2019-01-29

**Authors:** Katie Dunkley, Christos C Ioannou, Kathryn E Whittey, Jo Cable, Sarah E Perkins

**Affiliations:** 1 School of Biosciences, Cardiff University, Sir Martin Evans Building, Museum Avenue, Cardiff, UK; 2 School of Biological Sciences, University of Bristol, Bristol, UK

**Keywords:** cleaner fish, coral reefs, mutualism, personality, repeatability, trade-offs

## Abstract

Mutualistic interactions involve 2 species beneficially cooperating, but it is not clear how these interactions are maintained. In many mutualisms, one species interacts with multiple species, and since partners differ in terms of the commodities they trade, partner identity will directly influence the decisions and behaviors of interacting individuals. Here, we investigated the consequences of within and between-species diversity on a model cleaner–client interaction in a natural environment, by quantifying the behavior of both partners. We found that the predominant Caribbean cleaner fish, the sharknose goby (*Elacatinus evelynae*), shows personality variation as we documented repeatable individual differences in activity, boldness, and exploratory behaviors. Personality variation was associated with cleaner–client interactions: cleaner boldness and activity were significantly related to posing by clients and cleaning, respectively. Cleaner personality variation was also associated with the functional identity (sociality, mobility, body size, and trophic level) of clients posing and being cleaned. We thus demonstrate that partner identity can have consequences on mutualistic outcomes which will contribute to the context-dependency and highly heterogeneous patterns we observe at a population level. We also suggest that within- and between-species differences have consequences on partner choice, a feature that has been previously thought to be absent from these cleaner–client interactions.

## Teaser

Cleaner fish personality links with client identity to determine who interacts with whom and how. Our field study demonstrates that both client and cleaner identities contribute jointly to the variable patterns we observe for mutualisms. We challenge the existing paradigm that Caribbean cleaning gobies represent a “simple” system for studying cleaner–client interactions.

## INTRODUCTION

Mutualistic interactions, where 2 species beneficially cooperate, are observed in all ecosystems (Bronstein 2015), yet it is still not clear how these interspecific interactions are maintained. Mutualisms often involve food resources (e.g., nectar and ectoparasites) being traded for a beneficial act (e.g., pollination; Landry 2012, parasite removal; Arnal et al. 2001), known as service–resource interactions (Holland et al. 2005), but not all partners are equal in terms of the commodities they trade (Palmer et al. 2015). These interspecific interactions involve 2 individuals directly interacting at any one time, and thus the behaviors and traits of one partner, could directly influence the behaviors and traits of the other (Wolf and Weissing 2012). Partner identity will hence underpin the behavioral responses and decisions of animals during these cooperative interactions, influencing when individuals interact, with whom, and by how much (McAuliffe and Thornton 2015). Currently, our understanding of mutualisms is hypothesized to be context-dependent and highly heterogeneous (Bronstein 2015); so, investigating how individual partners influence mutualism outcomes will help to clarify the dynamics and hence evolution of mutualisms under natural conditions.

Within an environment, service providers only make up a small proportion of the biomass but interact with a disproportionately large number of other species (Sazima et al. 2010). As a result, mutualisms are often composed of networks of interacting species, with service providers carrying out ecosystem services, such as pollination (Landry 2012) and health enhancing parasite control (Clague et al. 2011; Waldie et al. 2011). Mutualists thus play a pivotal role in the structuring and functioning of ecological communities (Floeter et al. 2007; Sazima et al. 2010; Quimbayo et al. 2018). An iconic, well-studied service–resource mutualism, the cleaner–client interaction, is observed ubiquitously on coral reefs (White et al. 2007; Leung and Poulin 2008). The mutualism involves a cleaner removing ectoparasites and other material from the bodies of many client fish species (up to 132 different species; Grutter and Poulin 1998). Cleaning patterns, however, are inconsistent, with the same cleaner species showing preferences for different client types across studies. For example, cleaning gobies from the genus *Elacatinus* prefer larger clients in some studies (e.g., Whiteman and Côté 2002b; Grutter et al. 2005; Silvano et al. 2012), but not in others (e.g., Grutter and Poulin 1998; Arnal et al. 2000). These, like many other behavioral studies, focus on population patterns, which assume all conspecifics exhibit the same traits, or that variation around an average is random (Bolnick et al. 2011). Individuals within many invertebrate and vertebrate populations vary consistently in their behavior (also known as animal personality variation; Reale et al. 2007), and this variation can play a major role in shaping population-level patterns of species interactions and other ecological processes (Wolf and Weissing 2012). There are 5 recognized animal personality traits (Reale et al. 2007), and for many taxonomically distinct species, these traits can affect feeding and foraging behaviors. The personality traits boldness and exploration, for example, which can be broadly defined as an individual’s reaction to a risky (boldness) and new situation (exploration) (Reale et al. 2007), influence both an individual’s food intake and foraging success (Ioannou et al. 2008; David et al. 2011). Bolder and more exploratory individuals are expected to have increased metabolic demands since they are at an increased risk (e.g., to predation) and utilize the environment more widely (Careau et al. 2008; Brommer and Class 2017). A third personality trait, activity, which quantifies the general activity level of an individual (Reale et al. 2007), may also often predict foraging behaviors (Pruitt et al. 2012) as more active individuals are also expected to have increased energy demands (Careau et al. 2008; Brommer and Class 2017). Thus, personality traits, and their correlations with one another (forming a behavioral syndrome; Sih et al. 2012) are likely to play a role in food acquisition during mutualistic interactions: dedicated cleaners for example, gain all their nutrition from client derived material (Vaughan et al. 2017). Indeed, bolder cleaner fish (*Labroides dimidiatus*) have been shown to clean less honestly (i.e., cheat more) to acquire a more favorable reward (Wilson et al. 2014), while bolder black-billed magpie cleaner birds (*Pica pica*) interact with clients more frequently, facilitating greater access to protein-rich ticks (Found 2017).

However, the dynamics of mutualistic interactions are not just driven by a cleaner’s food dependency (Lenke 1988), because the resource provider’s behavior, engagement, and traits can also regulate outcomes of an interaction (Bever 2002; Bshary and Schäffer 2002). In cleaning interactions, clients can choose which cleaners to visit (Bshary and Schäffer 2002), and increase their chances of being cleaned (Côté et al. 1998), by presenting their body to cleaners (termed posing; Feder 1966). However, posing does not necessarily guarantee cleaning, and for some clients, they need not pose at all to be cleaned (Arnal et al. 2001; Dunkley et al. 2018). The cleaners past behavior towards the client can also influence their interactions with different cleaners: if a client has received a negative response from the cleaner, for example, they are less likely to revisit (Bshary and Schäffer 2002). Cleaners thus adapt their behaviors to ensure client satisfaction (Grutter and Bshary 2003). Partner feedbacks are hence an important component for maintaining positive interspecific interactions (Frederickson 2013), yet their role is largely ignored. Given that feedbacks can reinforce the development of behaviors (Houston and McNamara 1999; Sih et al. 2015), it would be expected that the expression of personality variation by cleaners would link with both the actor’s and receiver’s behavior. This prediction however has not yet been tested in a cleaning context, but personality variations have been shown to mediate other interaction types (e.g., predator–prey interactions; Pruitt et al. 2012, and service–service mutualisms; Schmiege et al. 2017). Client species differ in their propensity to engage in cleaning interactions (Côté et al. 1998; Bshary and Schäffer 2002), as well as the nutritional content that they represent to cleaners (Eckes et al. 2015). These differences mean that different clients will provide asymmetric benefits to the cleaning interaction. Larger (Poulin and Rohde 1997), group living and sedentary (Patterson and Ruckstuhl 2013) species, for example, are more prone to increased parasite loads. It is unknown whether individual cleaners respond asymmetrically to client identities and vice versa, influencing interaction patterns.

Here, to investigate the consequences of within and between-species diversity on the outcome of mutualistic interactions, we quantified both cleaner and client behavior in situ. We observed the cleaning interactions between the predominant Caribbean cleaner fish, the sharknose goby (*Elacatinus evelynae*), and their reef fish clients. These cleaner species rarely cheat by causing damage to client bodies (Soares et al. 2008), and thus their cleaning behavior represents a simpler system for studying cleaner–client interactions compared to the iconic bluestreak wrasse cleaners (*L. dimidiatus*, Côté and Soares 2011). Previous work has documented personality variation in (noncleaning) goby species (e.g., Magnhagen et al. 2014; Moran et al. 2016; Vallon et al. 2016), and as such, we expected sharknose gobies to show individual variation in major axes of personality traits (activity, boldness, and exploration). As personality traits can influence foraging behaviors, and clients will differ in the food material they host, we then determined whether different personality variations had consequences on cleaning behaviors (frequency, rate and which clients’ cleaners interacted with). Finally, since clients can also regulate mutualistic outcome patterns, we tested whether clients interacted differently with cleaners based on the cleaners’ personality traits (posing frequency, rates, and client functional identity).

## METHODS

### Identifying individual cleaner fish

Sharknose goby (*E. evelynae*) behavior was observed on Booby Reef, Man O’ War Bay, Tobago (11°19.344′N 060°33.484′W) over a 2-week period in June to July 2017 by daily snorkeling between the hours of 07:00 and 17:30. This study took place in the last 2 weeks of a 6-week field season, and thus gobies were assumed to be habituated to human presence on the reef. The small section of the reef sampled (60 × 70 m; reef depth: 1–2 m) is composed predominantly of algae covered dead elkhorn coral (*Acropora palmata*) and living brain coral (*Diploria* spp.). Individual sharknose gobies show strong site fidelity to their brain coral cleaning stations (Whiteman and Côté 2002a; Harding et al. 2003), so individuals (*n* = 17) could be repeatedly identified based on their cleaning station. The cleaning stations used in this study have been monitored annually as part of a long-term study (9 years, 2010–2018) and are located at least 1 m apart from one another. Cleaning interactions do not differ spatially on the reef (unpublished data). Sharknose gobies have a high turnover rate on their cleaning stations (mean age < 50 days documented in White et al. 2007), so it was not possible to quantify personality variation of the same individuals across years. Where more than 1 goby occupied a station (up to 4 gobies), a focal was identified based on relative size differences and only one individual was chosen per station.

### Quantifying cleaner fish personality variation

**Figure 1:**
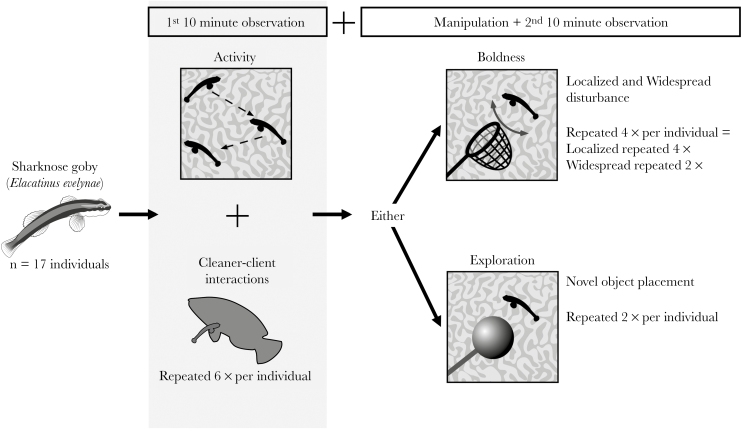
Methods for quantifying sharknose goby (*Elacatinus evelynae*) personality variation (activity, boldness, and exploration) in situ. Individual gobies were identified from their cleaning stations, and personality assays (boldness vs. exploration) were conducted on separate days using different stimuli. Activity quantification was carried out before each stimuli presentation. Recorded behaviors are listed in text and in Table 1.

Cleaner fish personality variation was quantified in situ at their fixed cleaning stations. Activity was determined through unmanipulated observations, while boldness and exploration were quantified using stimuli. To prevent habituation to the stimuli presented, boldness and exploration were quantified twice per stimulus (Figure 1). We did not test for individual variations in the aggressiveness and sociability axes of personality, since cleaning gobies are often found singularly or in small groups (Whiteman and Côté 2002a). All behaviors were recorded by observers and were not filmed due to the heterogeneous nature of the environment; cleaners often traverse around their large coral heads (ca. 1–2 m^3^) and thus could be regularly out of view from fixed cameras. Given that environmental variables, and the timescale between observations, can influence the consistency of behaviors (Bell et al. 2009; Wong et al. 2017; Pruitt et al. 2018), and here personality variations were quantified in the field, assays were repeated on consecutive days (where possible). The order of presenting the stimuli in the boldness and exploration assays were randomized across individuals; individuals experienced only one stimulus per day, and this occurred at a random time point. For all observations (*n* = 173), observers maintained a distance of 1.5 m from the cleaner.

**Table 1:** PCA loadings of behavioral variables used to generate first principal component scores (PC1) to quantity individual sharknose goby (*Elacatinus evelynae*) boldness, activity, and exploration scores

Personality trait	Recorded behaviors	PC1 loadings	Variation explained
Activity	Proportion of observation spent moving	0.508	47.22%
	Distance moved within observation	0.554	
	Speed	0.281	
	Frequency of movements that covered distance	0.564	
	Frequency of jerk movements	0.189	
	Frequency of open swims	0.012	
Boldness	Return time after disturbance by stick or net	−0.048	41.68%
	Difference in proportion spent moving pre- vs. postdisturbance	−0.589	
	Difference in distance moved pre- vs. postdisturbance	−0.546	
	Difference in speed pre- vs. postdisturbance	0.098	
	Difference in jerk frequency pre- vs. postdisturbance	−0.570	
Exploration	Time taken to return to position following novel object placement	−0.698	60.84%
	Time taken to be ≤ 20 cm from object	−0.701	
	Closest distance to novel object	−0.145	

### Activity

Activity (for *n* = 17 individuals) was quantified over a 10-min observation prior to a stimulus being presented in the boldness and exploration assays so that stimuli presentation did not interfere with quantifying activity (Figure 1). In contrast to the mid-water wrasse cleaners (e.g., *L. dimidiatus*), coral-dwelling sharknose gobies remain in direct contact with the coral at their cleaning stations (apart from when cleaning, and the occasional competition-induced move to adjacent coral; Whiteman and Côté 2002a; Côté and Soares 2011). Thus, within each observation, activity was measured as: the total 2D distance travelled by the cleaner across the coral surface or when swimming in open water, estimated to the nearest 5 cm (or to the nearest 1 cm if distance travelled <5 cm), the total duration of these movements, and the total duration of “jerk” movements (localized movement where the cleaner does not cover any distance over the coral head). To investigate behavioral consistency (Reale et al. 2007), activity was recorded up to 6 times (*n* = 6 for 12 individuals, *n* = 5 for 1 individual, and *n* = 2 for 4 individuals, dependent upon whether individuals were seen on their station, Figure 1).

### Boldness

The shyness–boldness axis of personality variation represents an individual’s reaction to a risky situation (Reale et al. 2007), so each focal cleaner (*n* = 15) was disturbed using both a localized and widespread disturbance for 20 s. Boldness behavior was based on 4 stimuli presentations; each cleaner was disturbed twice by both disturbance methods (localized vs. widespread, Figure 1, *n* = 14 individuals disturbed by both the methods; *n* = 1 individual only disturbed by a localized disturbance). The local disturbance involved proximally and distally moving a bamboo cane 10 cm from the focal cleaner, while a net (10 × 10 cm) was moved 1 m laterally to the coral head to create a widespread disturbance. The local method hence created a disturbance at the cleaner’s position on the station (representing a single client disturbing the cleaner), while the widespread method created a larger disturbance over the cleaner’s position and surrounding coral head (representing a shoaling client group disturbance). Typically, the focal cleaner quickly moved away from its position on the coral head during both disturbances. Following disturbance, we observed the fish for 10 min and recorded the time taken for the individual to return to the original predisturbance location. We also rerecorded the cleaner’s activity behavior within this 10-min observation as described previously. This protocol meant that an individual’s pre- versus postdisturbance movement behavior could be directly compared to quantify how an individual initially responded to a risky situation (similar to Houslay et al. 2018).

### Exploration

Exploration represents an individual’s reaction to a new situation (Reale et al. 2007), so cleaners (*n* = 16) were presented twice with a novel object (sand-filled orange ping-pong ball attached to a green garden cane weighted in a sand-filled bottle). The ball was placed 10 cm away from, but at the height of, the cleaner’s position on the station for 10 min and exploration was measured as the time taken for the cleaner to approach within 20 cm of the ball, the cleaner’s closest distance to the ball (to the nearest 1 cm if < 5 cm and to the nearest 5 cm if >5 cm away), and the time taken for the cleaner to return to its original location. Cleaners were observed inspecting and moving on the novel object. Observers placed the object as quickly and smoothly as possible to minimize and standardize any additional disturbance.

### Quantifying cleaner–client interactions

During the 10-min observations quantifying a cleaner’s activity behavior, we also recorded any cleaner–client interactions (Figure 1). Cleaning behaviors only took up a small proportion of the observation (mean ± SE = 10.6 s ± 2.09). We recorded the duration and frequency of cleaning of, and posing by, client species during each observation. Posing and cleaning rates, and frequencies within the observation, were used as a measure of client–cleaner behavior. The frequencies represent the total effort in cleaning or posing across all client species, while the rates are this effort per cleaning time (i.e., total cleaning frequency/total cleaning duration). Cleaners were not always in view to the observer due to the heterogeneous nature of their cleaning stations, thus the time a cleaner was out of view within each observation was accounted for by dividing frequency and duration data by the adjusted observation lengths.

### Data analysis

Data were analyzed using R, version 3.4.3 (R Core Team 2017). Generalized linear mixed models (GLMMs) were run using the lme4 package (Bates et al. 2015). For all models, response variables were rescaled from 1 to 10 using the scales package (Wickham 2017). This rescaling method does not remove the variability in responses, but simply transforms the small and/or negative variables to aid model fit. Cook’s distance identified influential points and models were rerun without them to determine their effect; influential points are only reported if they had a significant influence on the results. Model assumptions and fits, as specified by (Bolker et al. 2009) were assessed using residual plots and all continuous predictors were scaled and centered around zero to facilitate model convergence.

The behaviors measured during the activity trials were included in a principal component analysis (PCA) to produce an activity measure for each observation for each individual (*n* = 87 observations, *n* = 17 individuals); the same method was used to produce boldness (*n* = 56 observations) and exploration scores (*n* = 30 observations, behaviors and PC1 loadings listed in Table 1). Thus, multiple scores were calculated for each individual (max *n* = 6 scores for each individual for each personality trait). The first principal component score of each PCA was used as the measure of an individual’s boldness, activity, and exploration in each observation (as in Wilson et al. 2014). Table 1 shows the loading of each behavioral measure on each PC1 score. Prior to score calculation, behavior values were standardized using mean centering, and thus both negative and positive PC1 scores occurred.

To determine whether cleaners show personality variation in activity, boldness, and exploration, data were analyzed at an observation (i.e., per trial) level. GLMMs were used to analyze sharknose goby activity (Gamma family with inverse link function), exploration (inverse Gaussian family with inverse link), and boldness (inverse Gaussian family with inverse link, boldness scores were reverse transformed) scores from the PCAs. The following main effects were included within each behavioral trait analysis: day from the start of the study, time of day, time since last observation, number of cleaners at the station, disturbance order (based on presented order of 3 disturbances; cane, net, and novel object), replicate of each disturbance (for boldness and exploration: 1 or 2) and observer ID. For boldness, the disturbance method (cane vs. net) was also included as a main effect. Models were refined by removing the least significant term in each step. For all 3 GLMMs (activity, boldness, and exploration as the response variables), we included the random term of individual identity. For the best fitting models, likelihood ratio tests (LRTs) comparing models with and without this random effect of individual (similar to Houslay et al. 2018) determined whether among-individual differences existed in activity, boldness, and exploration scores.

To investigate the role of behavioral traits on cleaner–client interactions, an individual’s mean PC1 score was calculated separately for activity, boldness, and exploration behaviors. Pairwise correlations are widely used to investigate behavioral syndromes (Sih et al. 2012), and thus Spearman rank correlation tests determined whether individual sharknose gobies mean boldness, exploration, and activity scores related to one another in a behavioral syndrome. Individual sharknose goby mean activity, boldness, and exploration scores lie at different locations along continua, and thus for further analysis, we did not wish to exclude this between individual variation. As such, we used simple GLMs for all further analyses rather than nonparametric Spearman rank tests. The significance of main effects was determined by comparing models with and without the main effect.

To provide a measure of each cleaner individual’s cleaning interactions, an individual’s mean frequency and rate of cleaning and posing were also calculated from activity observations (i.e., before disturbances). Shannon’s diversity indices were calculated across all observations for each individual using the “vegan” R package (Oksanen et al. 2018) to provide a measure of the client diversity being cleaned and posing for each focal cleaner. Due to the small sample size (max = 17 individuals) and numerous predictors, forward stepwise GLMs determined whether an individual’s mean activity, boldness, and exploration behaviors predicted their mean cleaning frequency and rate (both Gamma family, log link), and diversity of clients cleaned (Gaussian family, log link). Predictors were initially kept in the model based on a critical α = 0.157 (Heinze et al. 2018), while the final model only contained significant predictors. The same method was used for mean posing frequency (Gamma family, log link), rate (inverse Gaussian family, log link), and diversity (Gamma family, log link). The main effects of mean activity, boldness and exploration, observer ID, and mean number of cleaners on the station were sequentially and manually added to the model based on descending correlation coefficients between each variable and the response variable. The main effects of cleaning frequency, rate and diversity cleaned, and posing frequency, rate and diversity posed were also added, where they were not considered as the response variable, to control for any feedbacks in behavior, since solicitation behaviors can initiate cleaning interactions.

To determine whether cleaner personality variation is associated with which clients are involved in and engage with cleaning interactions, client species were assigned maximum fork lengths using (Humann and Deloach 2014) and trophic levels using FishBase (Froese and Pauly 2018). A trophic level of 2 indicates an herbivorous client, while levels of 3 and above represent predatory clients. FishBase was also used to record clients as either solitary or gregarious (associate with > 3 individuals) and sedentary or free-ranging. Including all the clients, a sharknose goby cleaned, and their mean activity, boldness, and exploration scores, we used 3 GLM’s to determine whether sharknose goby behavioral traits predicted which clients were cleaned in terms of their size (inverse Gaussian family, inverse link), trophic level (2 vs. 3, binomial family, probit link), sociality (gregarious vs. solitary, binomial family, logit link), and mobility (free-ranging vs. sedentary, binomial family, probit link). A further 4 GLM’s determined whether posing client traits were predicted by activity, boldness, and exploration scores (size: inverse Gaussian family, log link, trophic level: binomial family, logit link, sociality: binomial family, cauchit link, mobility: binomial family, probit link).

## RESULTS

### Do cleaners show personality variation?

**Figure 2:**
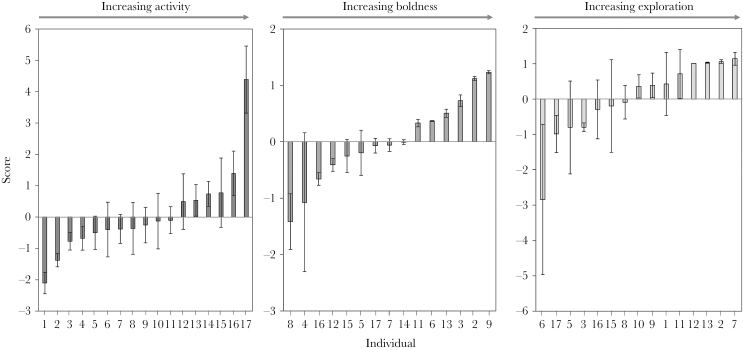
Mean (± SE) activity, boldness, and exploration scores (PC1; Table 1) for individual sharknose gobies (*Elacatinus evelynae*) occupying cleaning stations on Booby Reef Man O’ War Bay, Tobago. Individual activity scores are based on a maximum of 6 replicates, while boldness scores are calculated from 2 disturbance methods (cane and net) both repeated once, and exploration was quantified twice per individual.

Individual sharknose gobies (*E. evelynae*) differed from one another in their activity (LRT, χ^2^_1_ = 5.21, *P* = 0.022, final model _adj_*R*^2^ = 14.2%), boldness (LRT, χ^2^_1_ = 8.78, *P* = 0.003, _adj_*R*^2^ = 29.9%), and exploratory (LRT, χ^2^_1_ = 6.28, *P* = 0.012, _adj_*R*^2^ = 28.4%) behaviors, showing interindividual variation in these 3 traits (S.E. Min – Max across individuals: activity = 0.21–1.10, boldness = 0.07–1.98, exploration = 0.15–2.13; Figure 2). An individual’s mean boldness, activity, and exploration scores, however, did not significantly correlate with one another to form a consistent behavioral syndrome (between trait correlations: activity – bold *r*_s_ = −0.279, activity – exploration *r*_s_ = 0.036, boldness – exploration *r*_s_ = −0.071, all *P* > 0.10).

Although cleaners showed repeatable activity, boldness, and exploration behaviors, these traits were also affected by external factors. Cleaners were more exploratory as the time into day increased (GLMM, β = 0.06, χ^2^_1_ = 4.05, *P* = 0.044) and there was evidence for habituation as activity scores increased over the sampling period (GLMM, β = 0.03, χ^2^_1_ = 4.93, *P* = 0.026) and cleaners were bolder on the second replicate of each stimulus presentation compared with the first, irrespective of method (GLMM, β = 0.06, χ^2^_1_ = 5.84, *P* = 0.016), although boldness did decrease across the sampling period (GLMM, β = −0.10, χ^2^_1_ = 9.53, *P* = 0.002). Previous studies have documented social context influencing the expression of personality traits (Webster and Ward 2011; McDonald et al. 2016; Bevan et al. 2018), but here we found that the number of cleaners occupying a station did not affect personality scores (GLMMs, *P* > 0.05).

### Is personality variation associated with cleaner–client interactions?

**Figure 3:**
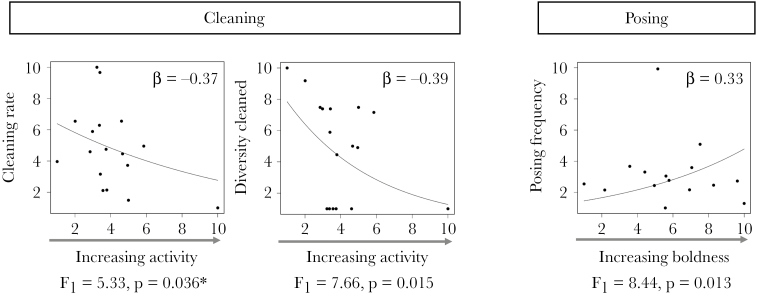
Significant GLM relationships between individual sharknose goby (*Elacatinus evelynae*) activity score and mean cleaning rate and diversity of clients cleaned, and boldness score and mean experienced posing frequency (all rescaled), with lines based on model coefficients. *P = 0.440 without influential point. Exploration scores did not predict cleaner–client interactions.

Through examining the 3 personality axes of activity, boldness and exploration, we find that these traits are linked to cleaner–client interactions. More active gobies cleaned a lower diversity of clients and cleaned at a lower rate, while bolder individuals experienced an increased posing frequency by their clients. Exploration had no effect on cleaner–client interactions (Figure 3).

We also found reciprocal positive feedbacks between individual cleaners’ cleaning frequencies and client posing frequencies across cleaners (GLMs: clean frequency – pose frequency: β = 0.41, *P* < 0.001, final model _adj_*R*^2^ = 46.3%; pose frequency – clean frequency: β = 0.57, *P* < 0.001, _adj_*R*^2^ = 68.8%). Client posing rates were also positively predicted by cleaners cleaning rates (GLM, β = 0.49, *P* = 0.002 _adj_*R*^2^ = 59.3%) and negatively related to cleaning frequencies (GLM, β = −0.48, *P* = 0.001). The diversity of posing clients also correlated positively with the diversity of clients cleaned and vice versa (GLM, diversity cleaned – diversity posed β = 0.41, *P* = 0.001, _adj_*R*^2^ = 64.8%, diversity posed – diversity cleaned β = 0.62, *P* < 0.001, _adj_*R*^2^ = 66.7%). Contrary to expectation, given the generally positive relationships between cleaner and client behavior, bolder individuals, who experienced an increased posing frequency did not clean more, and more active individuals which cleaned less frequently, did not experience more posing behavior (frequency and rate) from clients.

### Is cleaner personality variation associated with client traits?

**Figure 4:**
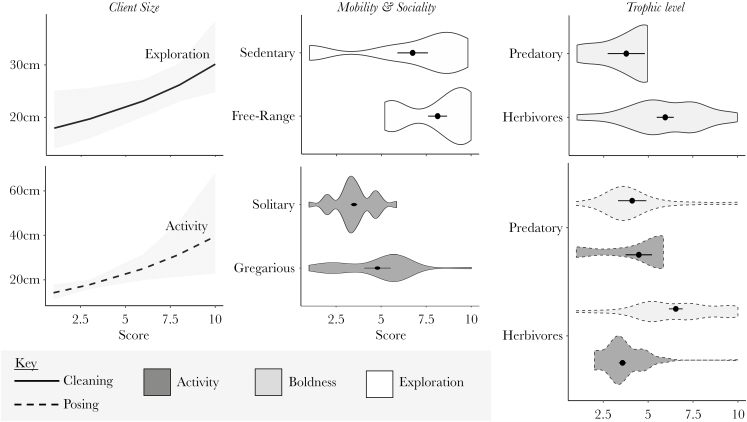
Significant associations from GLMs between sharknose goby (*Elacatinus evelynae*) boldness, exploration, and activity scores, and the clients cleaned (solid line) and posed (dashed line). PC1 scores (shown in Figure 1) were rescaled from 1 to 10 and mean scores were used in GLMs for each individual cleaner. Clients’ species are defined in terms of their functional traits: sociality, mobility, trophic level and body length (cm). Herbivores are defined as having a trophic level from 2 to 2.9, while predators represent the clients which have trophic levels >3. Line figures represents effects of mean activity, and exploration scores (from GLMs) across the range of client body sizes (min = 9 cm, max = 60 cm) observed posing and/or cleaned. Shaded regions show 95% CI. The outer shapes on the violin plot represent the range of mean personality variation scores over which different client types (sociality, mobility and trophic level) posed to and were cleaned by, different sharknose gobies. The thickness of each shape represents how frequently these client types posed to (dashed line) and were cleaned by cleaners with different activity, boldness and exploration mean scores. Point and lines show mean ± 95% CI.

Across our study, sharknose gobies cleaned 16 client species across 96 cleaning events, and cleaner personality variation was associated with which clients were cleaned. Bolder individuals cleaned herbivorous clients, while shyer gobies cleaned higher trophic level clients (Figure 4, GLM: χ^2^_1_ = 8.14, *P* = 0.004, final model _adj_*R*^2^ = 46.1%). Albeit low _adj_*R*^2^ values, individuals considered most exploratory cleaned larger clients (Figure 4, GLM: *F* = 4.67, *P* = 0.033, _adj_*R*^2^ = 4.9%) and the free-ranging fish (Figure 4, GLM: χ^2^_1_ = 7.27, *P* = 0.007, _adj_*R*^2^ = 8.3%). Cleaner activity did not influence which clients were cleaned (GLM: sociality, mobility, trophic level, and size all *P* > 0.10).

Seventeen client species posed for cleaners across 143 posing events with a total of 22 different species being involved in cleaner–client interactions. From a client’s perspective, larger fish posed for more active individuals (Figure 4, GLM: *F* = 13.03, *P* < 0.001, _adj_*R*^2^ = 8.2%), as did the more predatory species (Figure 4, GLM: χ^2^_1_ = 18.19, *P* < 0.001, _adj_*R*^2^ = 43.8%). The more predatory clients also posed for the shyer cleaners (Figure 4, GLM: χ^2^_1_ = 8.04, *P* = 0.005, _adj_*R*^2^ = 43.8%). Finally, solitary fish posed for less active cleaners over more active cleaners (Figure 4, GLM: χ^2^_1_ = 35.32, *P* < 0.001, _adj_*R*^2^ = 25.2%). Cleaner exploration did not associate with which clients posed to cleaners (GLM: sociality, mobility, trophic level, and size all *P* > 0.10).

## DISCUSSION

This field study demonstrates that sharknose goby (*E. evelynae*) cleaners show personality variation with consistent interindividual variation in their activity, boldness and exploration behaviors. Both activity and boldness were linked with cleaner–client interactions: more active cleaners cleaned a lower diversity of clients at a lower rate, while bolder individuals experienced an increased posing frequency by their clients. Personality variation was associated with client functional traits (sociality, mobility, trophic level, and body size), influencing which client species interacted with an individual goby of a given personality type. In summary, we show that within and between-species diversity has consequences on mutualistic outcomes.

Personality variation in activity influenced goby cleaner–client interaction dynamics. Due to increased metabolic demands, more active individuals are expected to increase their foraging behavior (Careau et al. 2008; Brommer and Class 2017), but here, more active individuals cleaned at a lower rate, and cleaned a lower diversity of clients. For other cleaner species, active behaviors (e.g., dancing; Youngbluth 1968, clapping; Chapuis and Bshary 2010, and rocking; Becker and Grutter 2005) attracts clients, but here the most active cleaners were not visited more frequently by client fish, suggesting gobies do not use obvious advertising movements. Given that sharknose goby cleaners gain all their nutrition from client derived material (Vaughan et al. 2017), more active gobies are utilizing a more limited resource (reduced cleaning rate and diversity of clients cleaned) for foraging gains. Therefore, they could be more efficient cleaners, or else the trait would not be expected to persist. An increased cleaning efficiency may explain why larger fish posed for more active gobies. Larger bodied fish tend to host more parasites (Poulin and Rohde 1997), and will also gain a greater cost when posing: posing temporarily stops a client from foraging (Grutter et al. 2002) and larger fish have increased energy demands (Bachiller and Irigoien 2012). Clients can learn the identity of specific cleaners from past positive experiences (Bshary and Schäffer 2002) or from observing how other individuals have been treated by the cleaner (Bshary 2002), thus visiting more efficient cleaners could reduce a client’s costs associated with cleaning. Conversely, more active gobies may not need to be efficient since here they interacted with all client types: more active gobies would thus not be restricted in the types of food resources available. A future study comparing the diets (in terms of nutritional gains) between cleaner gobies with contrasting levels of activity would be useful for determining how important these traits are for goby fitness in a foraging context.

Boldness influences foraging behaviors across many species (Reale et al. 2007; Biro and Stamps 2008; David et al. 2011), but here bolder cleaners did not differ in their cleaning behavior (i.e., foraging rates/frequencies) compared to shyer fish (contrasting Wilson et al. 2014). Partner choice can facilitate cooperation (Noë 2001), and bolder individuals were visited more frequently by clients compared to shyer individuals. Bolder animals are greater risk takers by definition (Reale et al. 2007); bolder *L. dimidiatus* cleaners for example, take risks by cheating their clients more frequently than shyer fish (Wilson et al. 2014). Although in other interaction contexts, bolder individuals are more likely to initiate and lead conspecific interactions (Ioannou and Dall 2016), a beneficial trait for posing clients, bolder individuals may risk not interacting with, and appeasing, all clients. Instead, bolder fish may reduce their own energetic costs by only cleaning preferred clients for maximum benefit (facilitated by an increased abundance of client fish posing for them creating choice options). Indeed, bolder individuals only cleaned herbivorous clients which feed intensely on the benthos throughout the day (Hay 1997). Benthic feeding brings potential clients in direct contact with the mobile crustacean ectoparasites which are often consumed during cleaning (Arnal et al. 2001; Grutter 2002), thus these clients may host high parasite loads and hence food rewards.

Exploration tendency increases how efficiently individuals utilize environments (Brommer and Class 2017; Careau et al. 2008), and although exploration did not link with cleaning behavior (contrasting Wilson et al. 2014), more exploratory cleaners differed in which clients they cleaned (more exploratory individuals cleaned larger clients and the free-ranging fish). Larger clients are assumed to be prone to increased parasite loads (Poulin and Rohde 1997) and being more exploratory may enable cleaners to quickly find parasites over a larger surface area: exploration is a measure of speed with which an individual moves around a novel environment (Reale et al. 2007). In contrast, free-ranging clients are assumed to host fewer parasites compared to sedentary species (Patterson and Ruckstuhl 2013), and thus being more exploratory may also facilitate cleaners to find and exploit more patchily distributed food sources (Mathot et al. 2012).

Mutualisms are maintained by positive interactions between partners, and for clients interacting with a cleaner they pay a cost. Thus clients must be responded to beneficially for them to return (Bshary and Schäffer 2002). Although we found strong feedbacks between posing and cleaning behavior, this was not reflected at an individual level. Cleaning behaviors expressed towards clients by more active, more exploratory or bolder fish did not reflect client posing behavior and vice versa. The identities of clients cleaned versus those posed also did not align, with the exception of herbivorous fish posing to and being cleaned by bolder gobies. Cleaner gobies are thought to rarely cheat by causing damage to client bodies (Soares et al. 2008), but this selective strategy for certain clients, irrespective of who is posing, may represent a subtler form of dishonesty. Overall, through partner identity, choice, and behavior, sharknose gobies with certain personality variations may reduce the maintenance of the mutualism in terms of the positive feedback between cleaning and posing.

Mutualisms involve many different asymmetric partners interacting with one another, and here we show for the iconic cleaner–client interaction that within and between-species diversity can influence mutualism outcomes. We demonstrate that there are asymmetries in interaction outcomes between different individuals, which will create heterogeneous patterns at the population level, a common feature across studies of cleaner–client interactions. Here, within-species individual differences (of cleaners) linked with between-species differences (of clients), contributing to who interacts with whom. Sharknose goby cleaning interactions have often been regarded as simple cleaning interactions with cleaner and client behaviors having no consequences on the interacting partner (Soares et al. 2008; Côté and Soares 2011). However, through behavioral feedbacks, and the expression of differing traits, we suggest that partner behaviors and identities can strongly influence mutualisms, albeit in a subtler way than those observed for the bluestreak wrasse (*L. dimidiatus*). Ultimately, this work may be applied to aquaculture, where cleaner fish are currently inefficiently deployed to biologically control ectoparasites of farmed fish (see Rae 2002). Given that client identity is fixed in these systems, our study suggests that selecting cleaners based on their behavioral traits (as suggested by Powell et al. 2017) or altering personality types through training (e.g., Frost et al. 2007) may increase the efficiency of deployed cleaners.

## FUNDING

This research was funded by a Natural Environment Research Council GW4+ studentship to K.D. (NE/L002434/1).

We thank Patricia Turpin (President of Environment Tobago) for field facilities, and Dr Amy Ellison, Ryan Mohammed and Environment Research Institute Charlotteville (ERIC) for field support.

Author contributions: K.D., S.E.P., and J.C. designed the study; J.C. and S.E.P. conducted a pilot study; K.D. and K.E.W. collected the data; K.D. with C.C.I. conducted the statistical analyses; and K.D. drafted the manuscript; all authors contributed to manuscript revisions.

Competing Interests: We have no competing interests.

Data accessibility: Analyses reported in this article can be reproduced using the data made available by Dunkley et al. (2019).
